# Stromal NNMT overexpression as an independent prognostic biomarker in lung adenocarcinoma and breast carcinoma

**DOI:** 10.1093/carcin/bgag020

**Published:** 2026-03-30

**Authors:** Yanzhong Wang, Yiping Wu, Shuyang Zheng, Xiaotian Yan, Xingtong Yu, Yuzhen Gao, Shuduo Xie, Zhinong Jiang, Rui An, Guoli Li, Jun Yang, Qingchao Tong, Xinyou Xie, Jun Zhang

**Affiliations:** Department of Clinical Laboratory, Sir Run Run Shaw Hospital, Zhejiang University School of Medicine, Hangzhou, Zhejiang 310016, China; Key Laboratory of Precision Medicine in Diagnosis and Monitoring Research of Zhejiang Province, Zhejiang Provincial Department of Science and Technology, Hangzhou, Zhejiang 310016, China; Zhejiang Engineering Research Center for Intelligent Manufacturing of Clinical Diagnostic Equipment, Zhejiang Provincial Department of Science and Technology, Hangzhou, Zhejiang 310016, China; School of Medicine, Shaoxing University, Shaoxing, Zhejiang 312000, China; Department of Clinical Laboratory, Zhuji Second People’s Hospital, Zhuji, Zhejiang 311811, China; Department of Clinical Laboratory, Sir Run Run Shaw Hospital, Zhejiang University School of Medicine, Hangzhou, Zhejiang 310016, China; School of Medicine, Shaoxing University, Shaoxing, Zhejiang 312000, China; Department of Clinical Laboratory, Sir Run Run Shaw Hospital, Zhejiang University School of Medicine, Hangzhou, Zhejiang 310016, China; Key Laboratory of Precision Medicine in Diagnosis and Monitoring Research of Zhejiang Province, Zhejiang Provincial Department of Science and Technology, Hangzhou, Zhejiang 310016, China; Zhejiang Engineering Research Center for Intelligent Manufacturing of Clinical Diagnostic Equipment, Zhejiang Provincial Department of Science and Technology, Hangzhou, Zhejiang 310016, China; Department of Clinical Laboratory, Sir Run Run Shaw Hospital, Zhejiang University School of Medicine, Hangzhou, Zhejiang 310016, China; Department of Clinical Laboratory, Sir Run Run Shaw Hospital, Zhejiang University School of Medicine, Hangzhou, Zhejiang 310016, China; Key Laboratory of Precision Medicine in Diagnosis and Monitoring Research of Zhejiang Province, Zhejiang Provincial Department of Science and Technology, Hangzhou, Zhejiang 310016, China; Zhejiang Engineering Research Center for Intelligent Manufacturing of Clinical Diagnostic Equipment, Zhejiang Provincial Department of Science and Technology, Hangzhou, Zhejiang 310016, China; Department of Surgical Oncology, Sir Run Run Shaw Hospital, Zhejiang University School of Medicine, Hangzhou, Zhejiang 310016, P.R. China; Department of Pathology, Sir Run Run Shaw Hospital, Zhejiang University School of Medicine, Hangzhou, Zhejiang 310016, China; Department of Clinical Laboratory, Sir Run Run Shaw Hospital, Zhejiang University School of Medicine, Hangzhou, Zhejiang 310016, China; Key Laboratory of Precision Medicine in Diagnosis and Monitoring Research of Zhejiang Province, Zhejiang Provincial Department of Science and Technology, Hangzhou, Zhejiang 310016, China; Zhejiang Engineering Research Center for Intelligent Manufacturing of Clinical Diagnostic Equipment, Zhejiang Provincial Department of Science and Technology, Hangzhou, Zhejiang 310016, China; Department of Clinical Laboratory, Sir Run Run Shaw Hospital, Zhejiang University School of Medicine, Hangzhou, Zhejiang 310016, China; Key Laboratory of Precision Medicine in Diagnosis and Monitoring Research of Zhejiang Province, Zhejiang Provincial Department of Science and Technology, Hangzhou, Zhejiang 310016, China; Zhejiang Engineering Research Center for Intelligent Manufacturing of Clinical Diagnostic Equipment, Zhejiang Provincial Department of Science and Technology, Hangzhou, Zhejiang 310016, China; Department of Clinical Laboratory, Sir Run Run Shaw Hospital, Zhejiang University School of Medicine, Hangzhou, Zhejiang 310016, China; Ningbo Diagnostic Pathology Center, Ningbo Municipal Health Commission, 685 North Huancheng Road, Ningbo, Zhejiang 315010, China; Department of Pathology, Ningbo Medical Center Lihuili Hospital, 57 Xingning Road, Ningbo, Zhejiang 315040, China; Department of Clinical Laboratory, Sir Run Run Shaw Hospital, Zhejiang University School of Medicine, Hangzhou, Zhejiang 310016, China; Key Laboratory of Precision Medicine in Diagnosis and Monitoring Research of Zhejiang Province, Zhejiang Provincial Department of Science and Technology, Hangzhou, Zhejiang 310016, China; Zhejiang Engineering Research Center for Intelligent Manufacturing of Clinical Diagnostic Equipment, Zhejiang Provincial Department of Science and Technology, Hangzhou, Zhejiang 310016, China; Department of Clinical Laboratory, Sir Run Run Shaw Hospital, Zhejiang University School of Medicine, Hangzhou, Zhejiang 310016, China; Key Laboratory of Precision Medicine in Diagnosis and Monitoring Research of Zhejiang Province, Zhejiang Provincial Department of Science and Technology, Hangzhou, Zhejiang 310016, China; Zhejiang Engineering Research Center for Intelligent Manufacturing of Clinical Diagnostic Equipment, Zhejiang Provincial Department of Science and Technology, Hangzhou, Zhejiang 310016, China; Department of Clinical Laboratory, Sir Run Run Shaw Hospital, Zhejiang University School of Medicine, Hangzhou, Zhejiang 310016, China; Key Laboratory of Precision Medicine in Diagnosis and Monitoring Research of Zhejiang Province, Zhejiang Provincial Department of Science and Technology, Hangzhou, Zhejiang 310016, China; Zhejiang Engineering Research Center for Intelligent Manufacturing of Clinical Diagnostic Equipment, Zhejiang Provincial Department of Science and Technology, Hangzhou, Zhejiang 310016, China

**Keywords:** NNMT, tumor stromal cell, independent prognostic biomarker, lung adenocarcinoma, breast carcinoma

## Abstract

This study investigated the expression profile and prognostic significance of nicotinamide N-methyltransferase (NNMT) in pan-cancer stroma, with focused validation in lung adenocarcinoma (LUAD) and breast carcinoma (BC). Integrated analysis of Genotype-Tissue Expression and The Cancer Genome Atlas databases revealed tumor-specific NNMT mRNA upregulation in these malignancies. Subsequently, the relationship between NNMT mRNA expression levels and clinical parameters across multiple cancer types was examined, with particular emphasis on LUAD, BC, and CRC. Single-cell RNA sequencing data further revealed elevated NNMT expression specifically within stromal compartments, with fibroblasts exhibiting the highest NNMT expression levels among stromal cell populations. Protein-level validation using the Human Protein Atlas database, the National Cancer Institute's Proteomics Data Commons, and the quantitative immunohistochemistry of our independent clinical cohorts demonstrated elevated stromal NNMT protein expression, particularly in cancer-associated fibroblasts. Cox regression analysis and survival curve analysis established stromal NNMT expression as an independent prognostic factor for overall survival in LUAD and BC patients (*P* < .05). In conclusion, high stromal NNMT levels correlated with poor prognosis, establishing it as a promising biomarker for risk stratification in LUAD and BC.

## Graphical Abstract

**Graphical Abstract:**
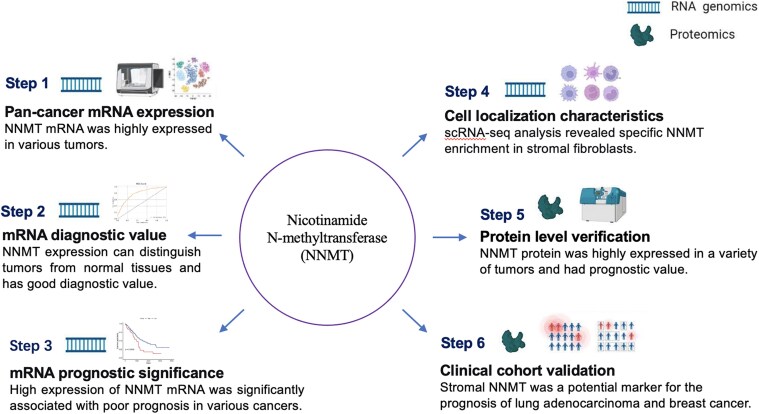

## 1. Introduction

The hallmark of metabolism activation as a tumor marker is outstanding [1]. Tumor occurrence and development are always associated with metabolic changes and undergo significant metabolic rewiring. This altered metabolism expedites the survival and proliferation of cancer cells, which have attracted a substantial amount of interest in cancer metabolism [2]. Cancer metabolism involved in the regulation of immune cells may lead to immune escape of tumor cells [3]. Despite extraordinary progress in the last decades, immune checkpoint therapy, which activates immune function by inhibiting checkpoints is still plagued by the tumor microenvironment and their understanding remains elusive [4, 5]. Therefore, identifying key metabolic regulators in the TME can help alter the process of tumor metabolic reprogramming and inhibit tumor development, serving as a promising strategy for improving cancer treatment.

Nicotinamide (NAM) N-methyltransferase (NNMT) was originally identified as a cytosolic enzyme that is responsible for the methylation of NAM and participates in the metabolism of NAD, playing a fundamental role in the regulation of multiple metabolic signaling pathways and remodeling cell epigenetic status [6-8]. Notably, in the last two decades, it was demonstrated that NNMT was known to be involved and upregulated in a wide variety of neoplasms [9, 10], such as breast cancer (BC) [11], colon cancer [12-15], gastric cancer [16-18], lung cancer [19], urinary system tumor [20, 21], skin cancer [22], glioblastoma [23], and so on. Preclinical research revealed that NNMT was correlated with the cancer cell migration and invasion and promoted the epithelial-mesenchymal transition. NNMT impacted the level of gene expression to modulate multiple pathways of cellular survival and apoptosis [24], which promoted the cancer phenotype [25], and played a key role in cancer progression with adverse clinical outcomes [25, 26]. In addition, NNMT-based therapies were proved to be a practical and effective treatment in anti-cancers [27, 28], especially the chemotherapy [11]. Consequently, a lot of research demonstrated NNMT might be a remarkable potential biomarker and therapeutic target for tumor diagnosis and treatment [26]. However, the mechanism of action of NNMT in pan-cancer has not been extensively studied, especially its role in metabolic regulation within the TME remains unclear.

According to the 2024 National Cancer Statistics, lung cancer and colorectal cancer (CRC) rank as the top two high-incidence cancers in China, while breast cancer remains the most frequently diagnosed malignancy among females. According to the global malignant tumor statistics report, lung cancer, breast cancer, and colorectal cancer are the top three high-incidence cancers in the world [29]. Previously, our research team has conducted in-depth research on the expression of NNMT in CRC [13]. Notably, we demonstrated that NNMT is highly expressed in CRC stromal cells and is significantly associated with advanced TNM staging, metastasis, and poor prognosis in CRC patients. This stromal expression pattern hints at a previously underappreciated role for NNMT in orchestrating the TME. However, to date, the specific location and function of NNMT in lung cancer have not been clearly determined. Non-small-cell lung cancer (NSCLC) constitutes over 85% of lung cancer cases [29], lung adenocarcinoma (LUAD) has a higher incidence than lung squamous cell carcinoma (LUSC) and become the main subtype of NSCLC [30]. Due to the development of various lung cancer therapies such as surgery, chemotherapy, radiotherapy, targeted therapy, and immunotherapy, the prognosis of lung cancer has been greatly improved [31]. However, LUAD has an insidious onset and lacks specific early clinical symptoms. Once diagnosed, most patients have already entered the advanced stage of the disease or have distant metastasis, and the prognosis is poor [32]. Therefore, the discovery of new lung cancer-related specific targets will benefit more LUAD patients. Although extensive research has demonstrated that NNMT is upregulated in various tumor tissues, its precise expression patterns and spatial localization remain to be fully elucidated [33]. While prior studies have explored the prognostic of NNMT expression in tumors, the spatial heterogeneity of NNMT protein expression, particularly its specific distribution within distinct anatomical microenvironments such as tumor cells and tumor stroma, and its association with clinical outcomes, still require in-depth investigation. Therefore, further studies are warranted to clarify the clinicopathological characteristics and prognostic value of NNMT expression in different compartments of LUAD and BC tumor tissues, thereby elucidating the mechanism of NNMT as a potential metabolic regulatory hub in the TME.

In the present study, we, firstly, comprehensively analyzed the NNMT gene and protein in multi-omics, revealing the landscape of NNMT relating to cancer metabolism regulation, tumor progression, and cell infiltration in pan-cancer by using well-known public databases. In addition, through the validation of LUAD and BC single-cell sequencing datasets and our clinical cohorts, we thoroughly clarified the high expression of NNMT in stromal cells. Moreover, the stromal NNMT (rather than tumor cell-expressed NNMT) acts an independent prognostic factor for overall survival in LUAD and BC patients, providing valuable insights into the tumor progression and metabolic regulation of LUAD and BC.

## 2. Materials and methods

### 2.1. Analytic pipeline

To fully access the expression of NNMT in human, the full data of TCGA were collected from previous data was collected in the Supplementary Table S1. In the present study, based on the multi-omics in the normal tissue and pan-cancer tissue samples (tissue and cell levels), the mRNA and protein levels of NNMT were comprehensively analyzed to reveal the roles in the tissue inflammation, cancer progression, tumor microenvironment, and clinical outcomes prediction.

### 2.2. Genotype-tissue expression (GTEx)

GTEx provides gene expression data from 17 382 RNA-seq samples across 54 tissue sites, which is publicly available in the UCSC (http://xena.ucsc.edu/). Given that, we can integrate the NNMT RNA expression of all sites to compare them in different tissue sites.

### 2.3. The cancer genome atlas (TCGA)

TCGA source data could be downloaded from the UCSC Xena data hubs (http://xena.ucsc.edu/). Here, 33 TCGA pan-cancer contained 10 536 samples with RNA expression profile data and their clinical data were collected to further analysis, including prognosis analysis, differential analysis between normal and tumor, or the correlation of NNMT with clinical stages of patients. Four prognosis endpoints, including overall survival (OS), disease-specific survival (DSS), disease free interval (DFI), and progression-free interval (PFI), were used to detect the relationship of NNMT RNA expression with prognosis in different cancers. The follow-up endpoint was defined as 31 December 2024.

### 2.4. Cancer cell line encyclopedia (CCLE)

CCLE is cancer cell lines source, freely accessible database, which has generated large amounts of next-generation sequence data, including DNA, RNA, protein, and DNA methylation for over 1100 cell lines across from 1000 cancer model (https://sites.broadinstitute.org/ccle/). The RNA-seq data of them were downloaded from the UCSC Xena data hubs for NNMT RNA expression and drug estimated analysis [34, 35]. According to the tissue of cell origin, we divided these cell lines into 24 categories which listed in the supplemental files.

### 2.5. Metabolism features

NNMT was a metabolic enzyme. So, we investigated the landscape of metabolism for NNMT in Pan-cancer. A total of 80 metabolism pathways were collected from the previous study, and then used the method of GSVA to calculate the enrichment scores for each patient in TCGA RNA-sequence data. We then got the relationship coefficient of them with NNMT RNA expression and their *P*-value as the input parameters in Pan-cancer. Similarly, the average coefficient above 0.3 with *P* < 0.05 and the number of them > 10 were regarded as significant pathways related to NNMT.

### 2.6. xCell analysis

xCell is an R package or webtool that uses cell type enrichment analysis from gene RNA expression data for 64 cell types gene signatures in tissue. It contained five main cells [myeloid, stroma, epithelial, lymphoid, hematopoietic stem cell (HSC)]. Pre-calculated TCGA data by xCell could be available in the website (https://xcell.ucsf.edu/).

### 2.7. Kyoto encyclopedia of genes and genomes (KEGG) pathway analysis

In terms of KEGG pathways analysis, we used the clusterProfiler R package to perform the gene set enrichment analysis for the co-expression RNA gene with NNMT to detect the related KEGG pathways based on the genes with their relationship coefficient with NNMT and their *P* values. The average normalized enrichment score (NES) for KEGG pathways for NNMT in pan-cancer, which was above 0.5 with a false discovery rate adjusted *P*-value < .05, was considered as significant pathway-related NNMT.

### 2.8. Proteins analysis

The National Cancer Institute's Proteomics Data Commons (PDC) is to make cancer-related proteomic datasets easily accessible to the public (https://pdc.cancer.gov/pdc/). Under the filter function in the website, we downloaded the 11 datasets with protein expression matrix from 10 types of cancer, including breast adenocarcinoma, colon adenocarcinoma (COAD), clear cell renal cell carcinoma, glioblastoma multiforme, head and neck squamous cell carcinoma, LUAD, LUSC, pancreatic ductal adenocarcinoma (PDAC), uterine corpus endometrial carcinoma (UCEC), and liver hepatocellular carcinoma (LIHC). Totally, NNMT protein expression from the 1971 tissue samples was used to compare the differences between normal and tumor proteomic. Among them, 1342 patients with prognosis data were used to explore the relationship of overall survival with NNMT protein. These data were listed in the supplemental files. In addition, to check the cells distribution of protein of NNMT, the results of immunofluorescence assay were collected form the Human protein atlas (HPA) databases (https://www.proteinatlas.org/ENSG00000166741-NNMT/tissue).

### 2.9. Single-cell analysis

To detect the distribution of NNMT in different cells, the Tumor Immune Single-cell Hub (TISCH, https://tisch.compbio.cn/home/), a single-cell RNA database, which collects 79 tumor datasets with single-cell level cell-type annotation, was used to evaluate the NNMT RNA expression in each cell type in the pan-cancer [36]. To integrate batch effects among multiple single-cell sequencing datasets, the Seurat software package was utilized for data integration, and the Harmony algorithm was employed to remove batch effects by applying it on the first 30 principal components. Malignant cells, immune cells and stromal cells were the main three type in the database. The 22 mini cell types were also annotated to the single cells for these datasets.

### 2.10. In-house patients collection and follow-up

This retrospective study analyzed two independent cohorts: (i) 159 consecutive patients with LUAD who underwent R0 resection at Taizhou Municipal Hospital (2012–2018) and (ii) 90 patients with primary breast cancer treated with mastectomy at Sir Run Run Shaw Hospital (2011–2018). Patients were not randomly allocated to groups, but rather assigned based on their diagnosis and surgical treatment during the study periods, reflecting real-world clinical practice. The LUAD cohort had a median age of 64 years (range: 45–87) with comparable gender distribution (64 males vs 95 females), while the breast cancer cohort comprised exclusively female patients with a median age of 53 years (range: 25–79).

All pathological diagnoses were independently verified by two blinded pathologists to minimize diagnostic bias, with discrepant cases resolved through consensus review. For LUAD cases, hematoxylin and eosin staining and immunohistochemical confirmation were performed by investigators blinded to clinical outcomes. Breast cancer specimens underwent similar blinded histopathological evaluation.

The study employed multiple strategies to minimize bias: pathological assessments were conducted independently by two investigators blinded to patient outcomes, and statistical analyses were performed by researchers unaware of group allocations. Inclusion criteria required: (i) histopathological confirmation, (ii) complete clinicopathological data, and (iii) written informed consent. Exclusion criteria eliminated patients with distant metastasis, prior malignancies, or incomplete records.

Regular telephone follow-up was conducted until the study endpoint (31 December 2024), during which five LUAD patients and six Bc patients were lost to follow-up. All remaining patients maintained complete follow-up data. The study protocol and use of patient materials were approved by the Ethics Committees of both institutions, with written consent obtained from all participants prior to inclusion.

### 2.11. Immunohistochemical staining and evaluation of tissue samples

All the LUAD, BC tumor, and their adjacent tissue samples were fixed in formalin and embedded with paraffin. Firstly, after deparaffinized, rehydrated, and antigen retrieval, normal goat serum was used to blocked the non-specific binding for 10 min at room temperature. In addition, after incubating the mouse monoclonal anti-human NNMT antibody (1E7, dilution 1:1400) that was prepared through the hybridoma technique as previously described [11] for 40 min, the slides were incubated with biotinylated goat anti-rabbit secondary antibody (Cat# BA-1000, RRID:AB_2313606) for 30 min. A diaminobenzidine Substrate Kit was used for chromogenic reaction. Finally, the images of IHC were captured by digital slide scanning system (KF-PRO-005).

NNMT protein expression was evaluated under double-blind conditions by two independent pathologists who had no access to the clinicopathological data. Scoring was performed separately for the tumor cell compartment and the stromal compartment: tumor cell compartment: Cytoplasmic staining in morphologically unequivocal malignant epithelial cells was scored. Areas of necrosis, keratinization, and ambiguous cells at the tumor-stroma interface were excluded; stromal compartment: Staining was evaluated primarily in the spindle-shaped, fibroblast-like cells [cancer-associated fibroblasts (CAFs)], and some immune cells within the stroma surrounding tumor nests. Vessels and normal smooth muscle structures were excluded. The classification of protein expression level was labeled as 0 (no staining), 1+ (weak staining), 2+ (moderate staining), or 3+ (intense staining). Finally, the percentage of positive cells and the respective intensity scores were integrated to calculate the staining score. The staining score had a minimum value of 0 and a maximum value of 300. The patients with different staining scores were classified as low and high expression groups according to the survimer R packages.

### 2.12. Ethics statement

This study was performed in line with the principles of the Declaration of Helsinki. The use of patient's information and tissues was sanctioned by the Ethics Committee of the Sir Run Run Hospital (NO. 20230422 and 20230427). All patients provided written informed consent before their inclusion in this study.

### 2.13. Statistical analysis

Statistical analyses, such as unpaired two group *t*-test, Wilcoxon rank-sum test, and Fisher exact test, were performed to test the differences between different groups of patients for NNMT and other clinical variables. To check the prognostic value of NNMT, then, we used the log-rank test, univariate and multivariate Cox proportional hazard regression to determine the independent risk factor for the patients in the different cohorts. The selection of variables for the Cox regression models in this study was driven by clinicopathological factors with established prognostic significance in LUAD and BC from prior research (e.g. TNM stage, age, gender, and key serum tumor markers, such as CEA, CA199, CA125, and AFP). These established factors were analyzed in conjunction with stromal NNMT to assess the independent prognostic value of NNMT after controlling for these traditional variables. Variables with *P* values < .1 in univariate Cox regression analysis were included in the multivariate Cox regression. Excluded special instructions, all statistical analyses were performed in R software (version 4.0) and all reported *P* values were two-sided and statistical significance was set at 0.05.

## 3. Results

### 3.1. NNMT mRNA had a high expression in pan-cancer and activated many metabolism pathways

**Figure 1:**
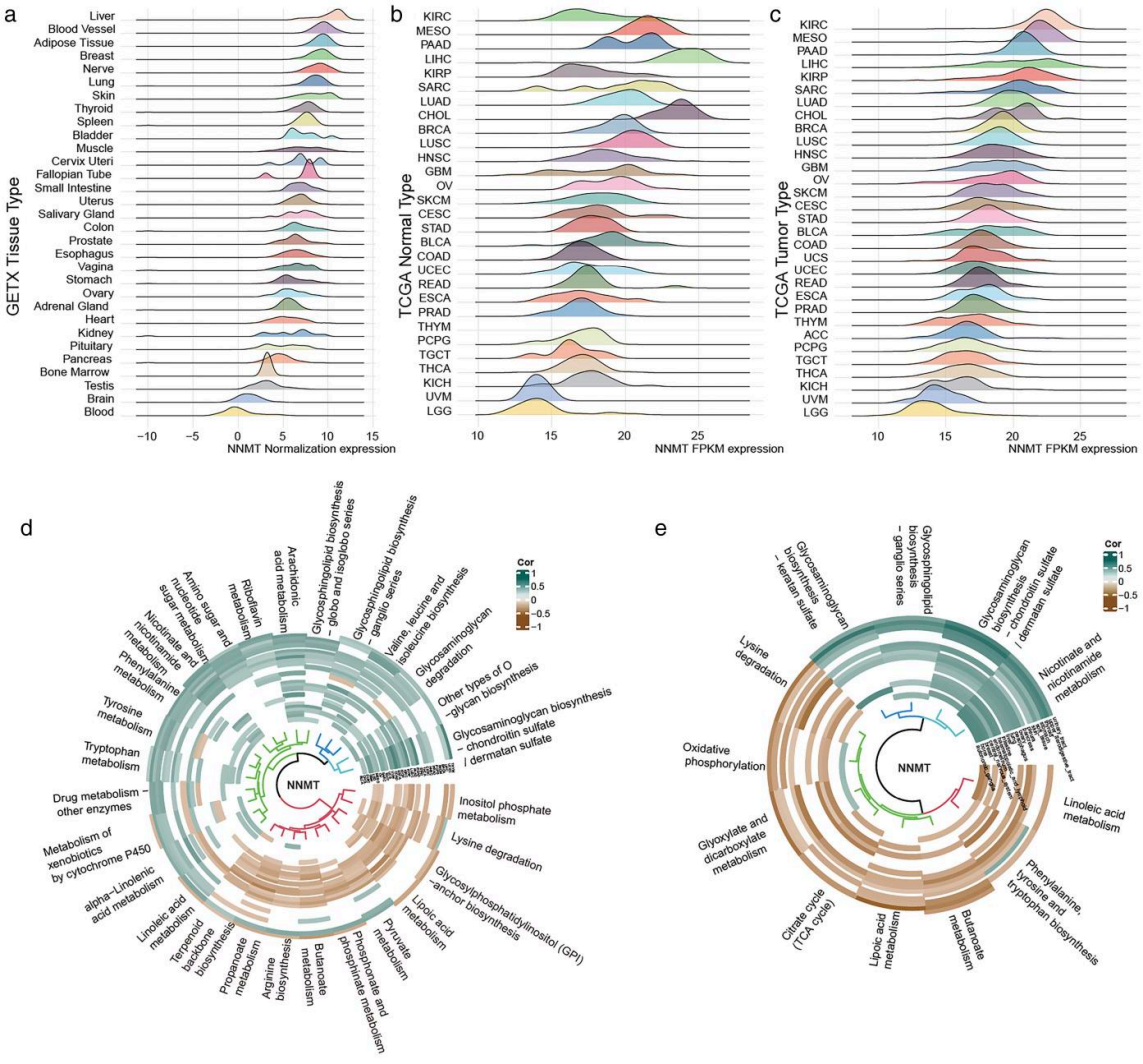
The specific tissue site of NNMT mRNA and its role in metabolism in Pan-cancer. (a) NNMT mRNA expression sort in normal tissue sites from GTEx database; (b) NNMT mRNA expression sort in normal tissues from TCGA database; (c) NNMT mRNA expression sort in normal tissues from TCGA database; (d) NNMT-related metabolism pathways in TCGA. (e) NNMT-related metabolism pathways in CCLE.

Firstly, we showed the distribution of NNMT expression in the body tissue in the GTEx dataset, which contained 24 types of tissue sites in the normal. It revealed that NNMT mRNA was preferentially expressed in specific tissues, such as the liver, blood vessels, and adipose tissue (Fig. 1a). In terms of the TCGA cohort, we showed the ranking of NNMT expression in the 29 adjacent tissues and 31 tumor tissues with the ridge plot. Then, they still found that the tissue from the hepatobiliary and urinary systems had high NNMT mRNA expression levels (Fig. 1b and c), such as LIHC, PAAD, KIRC, and CHOL. Of particular clinical relevance, high NNMT expression was also observed in LUAD, BRCA, and COAD, which represent three of the most incident and mortality-associated malignancies in the Chinese population. The mean expression data were listed in the Supplementary Table S2. As NNMT was a metabolism enzyme and expressed high in the liver, we found that it regulated many metabolism pathways. In the TCGA cohorts, we got 27/80 NNMT-related metabolism pathways. Among them, 10 out of 27 were negative ways, and 17 out of 27 were positive ways (Fig. 1d). Similarly, 12 out of 80 metabolism pathways were regarded as NNMT-related pathways at the Cell level from the CCLE database (Fig. 1e). Among them, four metabolism pathways were positively related to NNMT. The other eight metabolism pathways were negatively associated with NNMT. At both the tissue and cellular levels, glycosphingolipid biosynthesis, glycosaminoglycan biosynthesis, and Nicotinate and nicotinamide metabolism were positively related to NNMT, and lipoic acid metabolism, butanoate metabolism, and linoleic acid metabolism were negatively associated with NNMT.

### 3.2. High NNMT mRNA expression affected the prognosis of patients with pan-cancer

**Figure 2:**
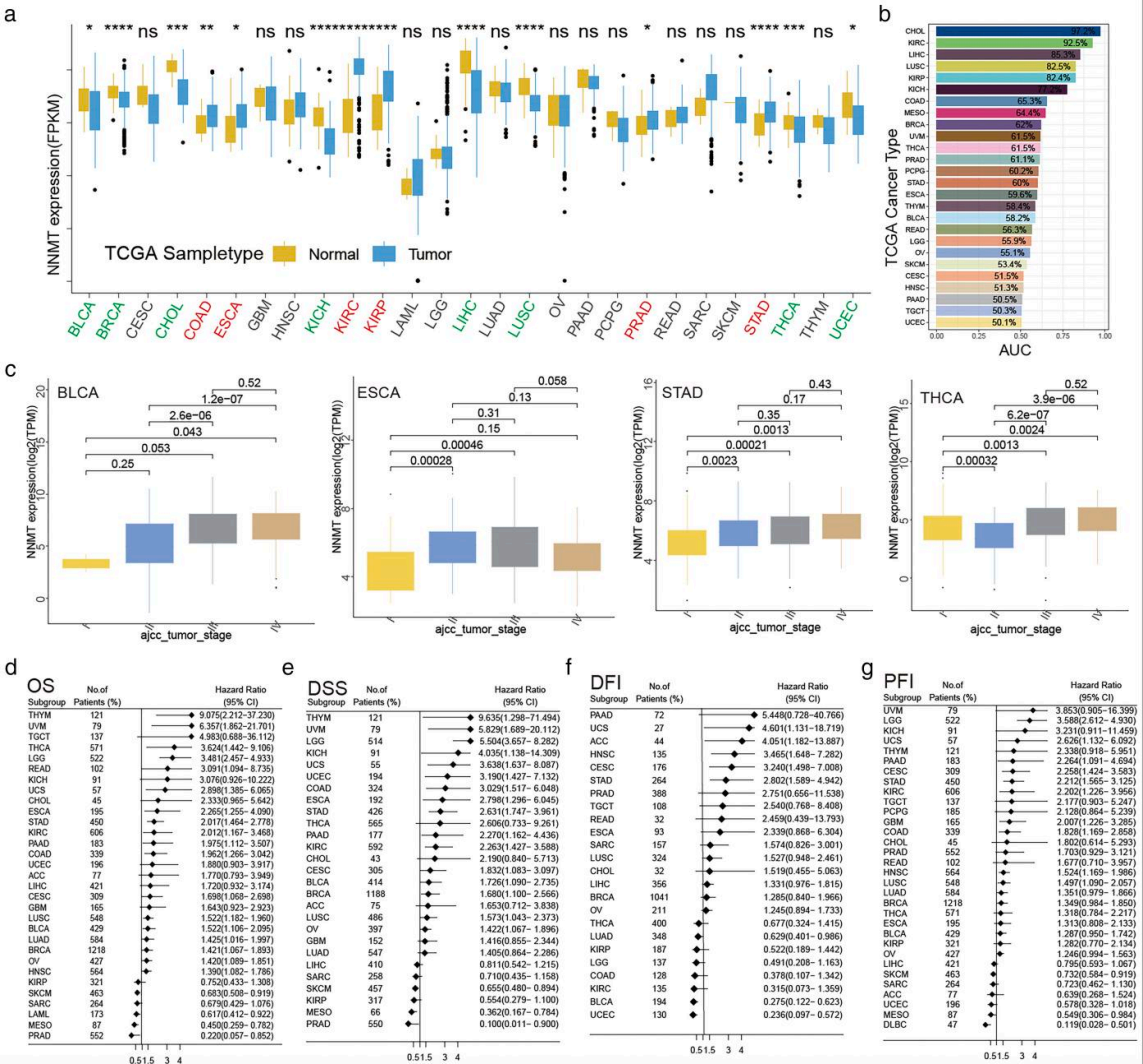
The characteristic of NNMT mRNA expression in Pan-cancer. (a) The differences of NNMT mRNA expression between normal and tumor tissue for TCGA database (red: high in the tumor, green: low in the tumor). (b) The AUC of ROC of NNMT in tumor diagnosis in the TCGA database. (c) The relationship of NNMT with some particular tumors in the TCGA database. The prognosis of NNMT mRNA expression in Pan-cancer. (d) OS; (e) DSS; (f) DFI; (g) PFI.

Because of the tumor heterogeneity, it has not the consistency of the differences in NNMT expression between the adjacent and tumor tissue in these tumors. Among them, tumor NNMT expressed higher than average tissue in COAD, ESCA, KIRC, KIRP, PRAD, STAD, but not in the BLCA, BRCA, CHOL, KICH, LIHC, LUSC, THCA, and UCEC (Fig. 2a). According to the receiver operating characteristic analysis, NNMT can have good diagnostic values for the 26/33 types of pan-cancer (Fig. 2b and Supplementary Table S3). In addition, we also found that the NNMT mRNA expression was also related to the clinical TNM stage in BLCA, ESCA, STAD, and THCA (*P* < .05, Fig. 2c).

In the TCGA pan-cancer cohorts, the univariate Cox regression was used to investigate the predictive values of NNMT mRNA expression for the four types of prognosis indexes of patients, OS, DSS, DFI, and PFI. We found that NNMT mRNA was significantly related to the OS of 22 out of 33 cancers, 18 out of 22 were poor, and the remaining four were good (Fig. 2d). In terms of the DSS (Fig. 2e), it had similar results for NNMT mRNA expression. However, for the duration of PFI and DFI, NNMT mRNA had significant prognostic values only in 13/32 and 8/28 cancers (Fig. 2f and g). NNMT mRNA expression had a poor prognosis in 10/13 and 5/8 cancers, respectively. All detailed data were listed in the Supplementary Table S3. These results show the consistency of these prognosis values for NNMT mRNA expression in the pan-cancer.

### 3.3. NNMT protein expression was closely related to the prognosis and clinical characteristics of pan-cancer

**Figure 3:**
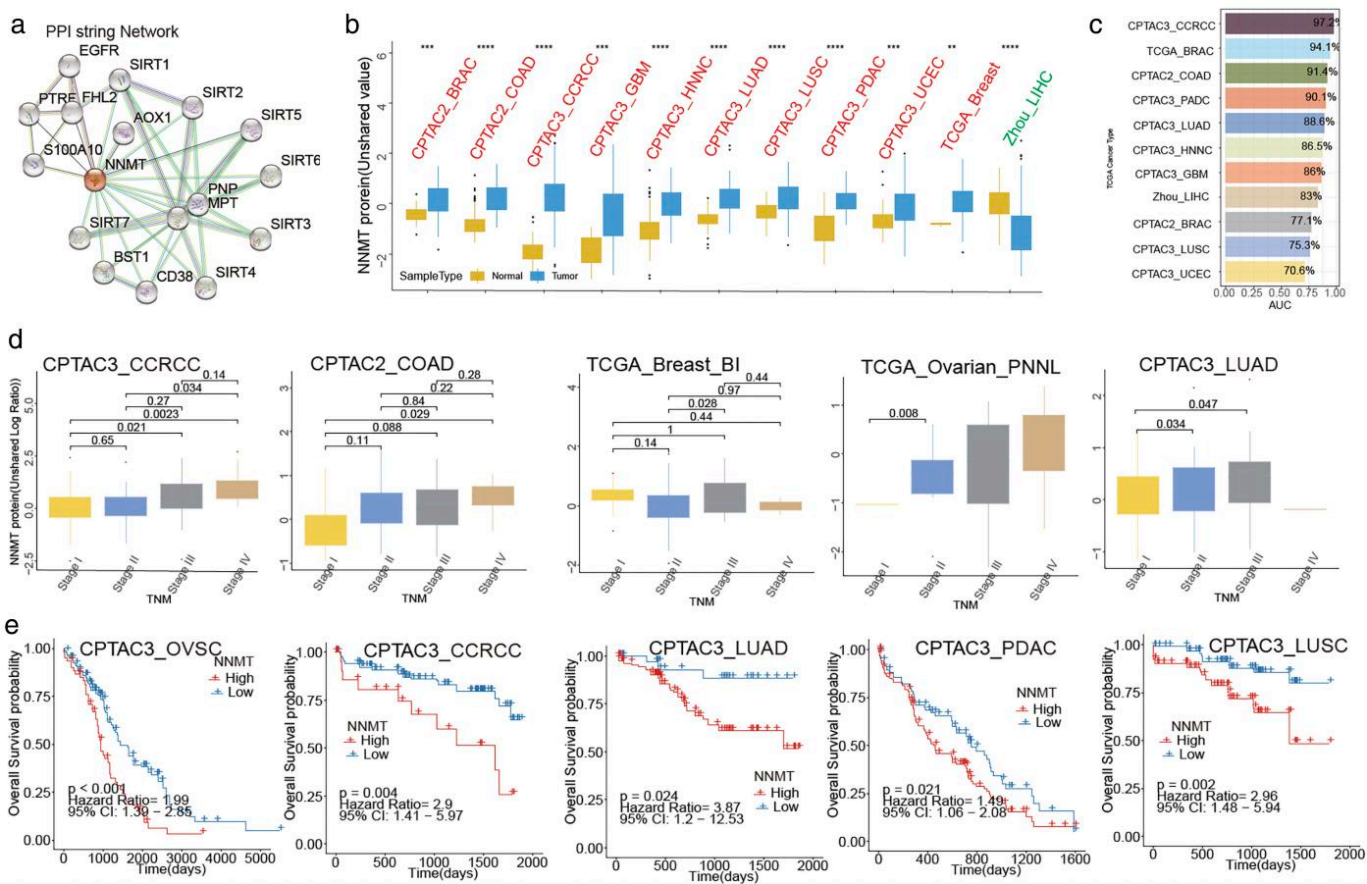
The protein features of NNMT in HPA, PPI, and PDC. (a) PPI String analysis for NNMT protein. (b) The differences in NNMT protein expression between normal and tumor tissue in PDC. (c) Diagnostic value of NNMT protein in pan-cancer according to ROC analysis. (d) Five types of cancers associated with NNMT protein expression and clinical stage in pan-cancer tissues. (e) Kaplan–Meier survival analysis of tumor types with statistically significant upregulation of NNMT protein expression.

Protein analysis will be helpful for us further to understand the complex functions of NNMT in tumors. We, first, chose two cell lines with Immunofluorescence in the HPA database, RH-30 and RPTEC TERT1, which showed that the NNMT protein location mainly gathers in the Golgi apparatus and Cytosol in the cells (Supplementary Figure S1). PPI analysis indicated that NNMT protein interacted with many proteins with critical biological functions, such as AOX1, SRRT family genes, BST1, CD38, MPT, and so on (Figure 3a). Then, we analyzed the protein expression in the 11 proteins expression profile data with 10 types of tumors (Supplementary TableS4). It was found that NNMT was higher in the tumor tissues than in normal or adjacent tissues in almost all cancers, except liver cancer (Fig. 3b and Supplementary Table S5). The high AUCs of NNMT also showed a vast difference between tumor and normal (Fig. 3c). Similar to the mRNA, the NNMT protein also was associated with the clinical TNM stage in RCC, COAD, BRCA, OV, and LUAD (all KS test *P* < .05, Fig. 3d). Lastly, it could discriminate the prognosis of some cancers, including LUAD, LUSC, OV, PDAC, and RCC (all log-rank test *P* < .05, Fig. 3e and Supplementary Table S5).

### 3.4. High expression of NNMT in stromal cells

**Figure 4:**
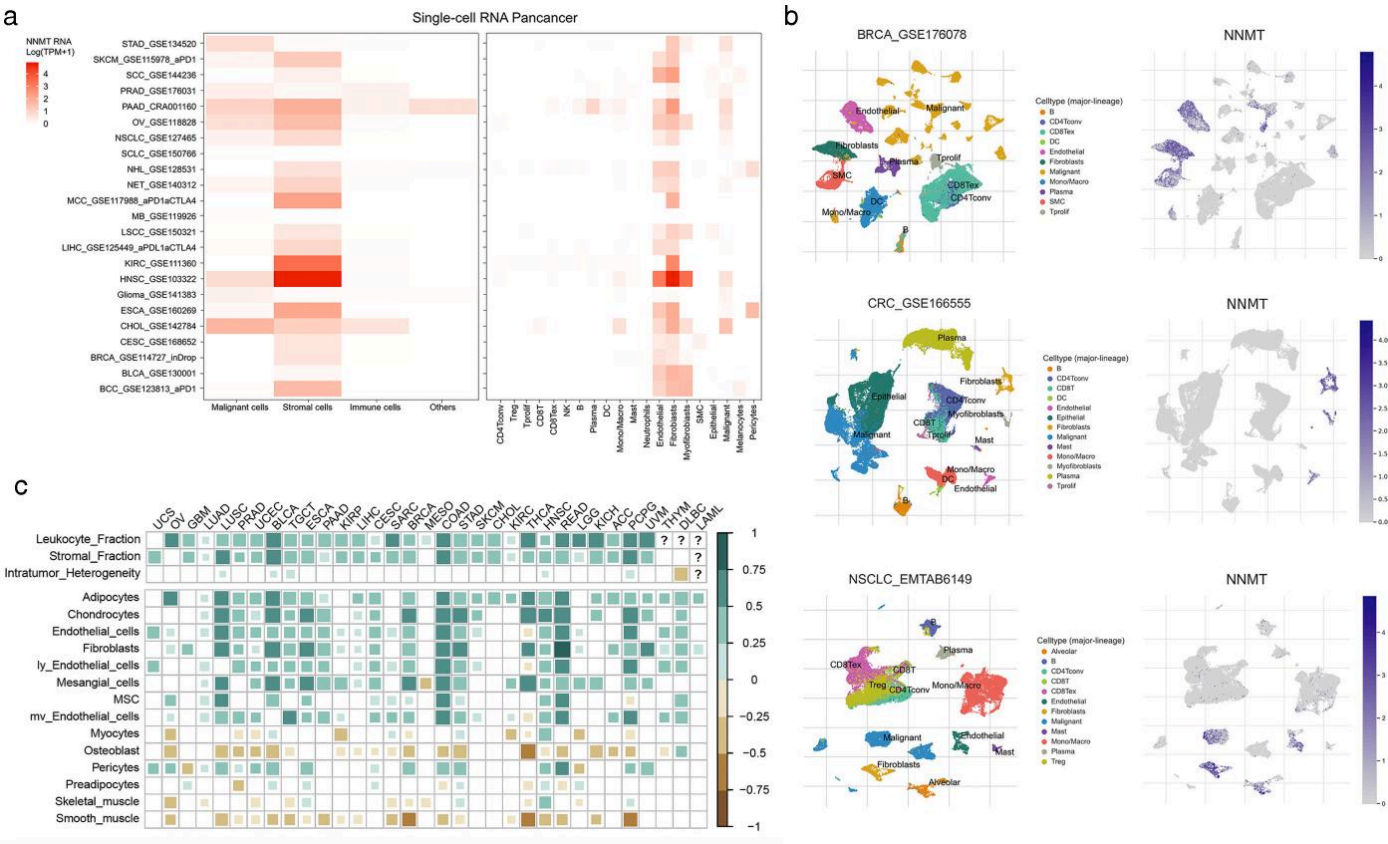
The distribution of NNMT at single-cell and tissue levels. (a) The expression of NNMT mRNA in 23 single-cell public datasets from TISCH. (b) The represented dataset for NNMT mRNA expression; (c) The relationship of NNMT mRNA expression with stroma-related cell types.

A total of 23 single-cell sequence profiles from TISCH were collected to estimate the cell distribution of NNMT at the single-cell level, and it suggested that NNMT is expressed higher in the stromal cells than in other cells. Among them, fibroblast cells’ NNMT expression is the highest compared with other cells (Fig. 4a). In detail, the fibroblast and endothelial cells have higher NNMT expression than other mini-type cells. BRCA_GSE176078, CRC_GSE166555, and NSCLC_EMBTAB6149 were representative datasets of BC, CRC, and LUAD, respectively, which confirmed that NNMT was highly expressed in interstitial cells in these three tumors (Fig. 4b). Also, NNMT RNA expression was strongly related to the stromal components, such as the adipocytes, chondrocytes, endothelial cells, and so on (Figure 4c). All the single data were listed in the Supplementary Table S6.

### 3.5. High expression of NNMT in LUAD and BC

**Figure 5:**
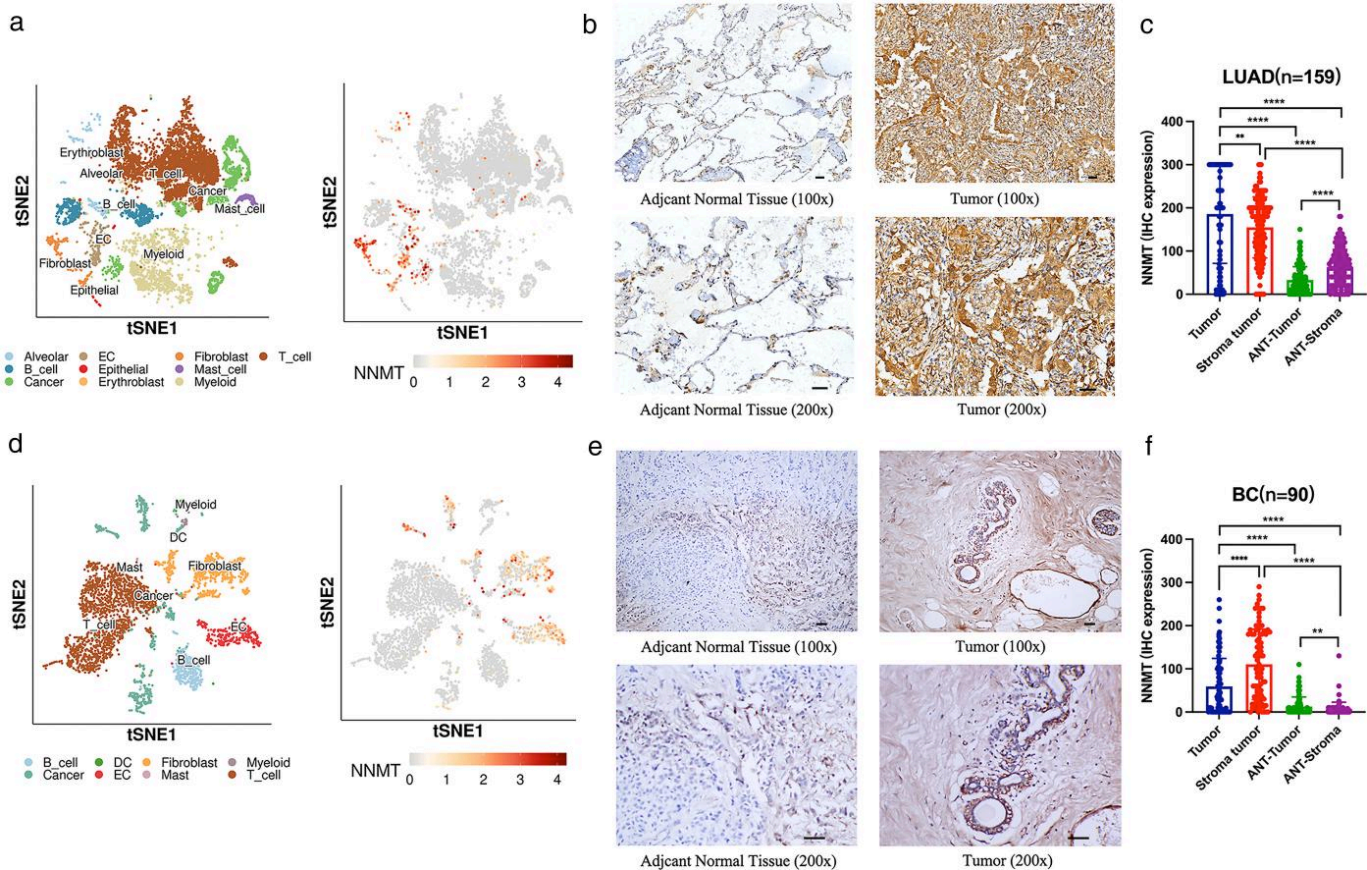
Verification of NNMT expression in LUAD and BC patients. (a) The tSNE plot of cell clustering and NNMT expression in the SCAR_Atlas_1138 dataset. (b) The represented IHC results for NNMT protein within 100 × and 200 × in LUAD tissues (scale bar = 100 μm). (c) The comparisons of NNMT among tumor, stromal tissue and other tissues in the LUAD (scale bar = 100 μm). (d) The tSNE plot of cell clustering and NNMT expression in the SCAR_Atlas_0924 dataset. (e) The represented IHC results for NNMT protein within 100 × and 200 × in BC tissues (scale bar = 100 μm). (f) The comparisons of NNMT among tumor, stromal tissue and other tissues in the BC.

The distribution of NNMT in LUAD cell populations was analyzed using the SCAR_Atlas_1138 single-cell sequencing dataset (Fig. 5a). It was found that NNMT was mainly enriched in stromal cells such as fibroblasts and endothelial cells. To verify the expression of NNMT in LUAD, we collected a LUAC cohort (*n* = 159) and performed IHC to evaluate the expression of NNMT protein in cancer tissues and corresponding adjacent tissues. The baseline characteristics of LUAD were listed in the Table 1. We found that in a large number of specimens, NNMT protein expression was localized in the cytoplasm of cancer cells, and was also expressed in tumor stromal cells, and NNMT was lowly expressed in adjacent tissues (Fig. 5b). NNMT in tumors and tumor-associated stroma was significantly higher than that in adjacent tissues (Fig. 5c). Moreover, NNMT expression in tumor-associated stroma significantly correlated with TNM, tumor size, and distant metastasis of LUAC (Table 1).

**Table 1:** The clinical characterize of the LUAD patients in a clinic cohort (*n* = 159).

Clinical parameters	Overall	Low NNMT group	High NNMT group	*P*
***n***	159	80	79	
**Age [mean (SD)]**	63.90 (8.18)	64.30 (8.87)	63.58 (7.63)	.911
**Sex (%)**				
** Male**	64 (40.3)	32 (40.0)	32 (40.5)	.949
** Female**	95 (59.7)	48 (60.0)	47 (59.5)	
**Differgrade (%)**				.166
** 1**	39 (24.5)	16 (20.0)	13 (16.5)	
** 2**	91 (57.2)	44 (55.0)	47 (59.5)	
** 3**	14 (8.8)	9 (11.3)	5 (6.3)	
** N/A**	12 (7.5)	10 (12.5)	2 (2.5)	
** Tis**	3 (1.9)	1 (1.2)	2 (2.5)	
**TNM stage (%)**				**<**.**001**
** I**	118 (74.2)	50 (86.3)	68 (86.1)	
** II**	7 (4.4)	6 (2.5)	1 (1.3)	
** III**	15 (9.4)	10 (6.3)	5 (6.3)	
** IV**	16 (10.1)	13 (5.0)	3 (3.9)	
** 0**	3 (1.9)	1 (0.0)	2 (2.5)	
**T (%)**				.**048**
** T0**	3 (2.1)	1 (1.3)	2 (2.5)	
** T1**	80 (55.9)	34 (42.5)	46 (58.2)	
** T2**	56 (39.2)	28 (35.0)	28 (35.4)	
** T3**	2 (1.4)	2 (2.5)	0 (0.0)	
** T4**	2 (1.4)	2 (2.5)	0 (0.0)	
***N* (%)**				.240
** N0**	120 (83.3)	54 (67.5)	66 (83.5)	
** N1**	24 (16.7)	14 (17.5)	10 (12.6)	
**M (%)**				.**009**
** M0**	143 (89.9)	67 (83.8)	76 (84.8)	
** M1**	16 (10.1)	13 (16.2)	3 (15.2)	
**CA199(%)**				.076
** <37 ng/ml**	147 (92.5)	75 (93.8)	72 (96.2)	
** ≥37 ng/ml**	12 (7.5)	5 (6.2)	7 (8.9)	
**CEA (%)**				.094
** <8 ng/ml**	133 (83.6)	68 (85.0)	65 (82.3)	
** ≥8 ng/ml**	26 (16.4)	12 (15.0)	14 (17.7)	
**AFP (%)**				.154
** <20 ng/ml**	157 (98.7)	79 (98.8)	78 (98.7)	
** ≥20 ng/ml**	2 (1.3)	1 (1.2)	1 (1.3)	
**CA125 (%)**				.434
** <35 ng/ml**	144 (90.6)	76 (95.0)	68 (86.1)	
** ≥35 ng/ml**	15 (9.4)	4 (4.0)	11 (13.9)	
**NNMT expression**				
** Stroma of tumor [mean (SD)]**	172.11 (63.56)	97.00 (46.73)	213.29 (31.45)	**<**.**001**

Clinical characteristics of 159 LUAD patients stratified by NNMT expression (low vs. high). Significant differences were observed in TNM stage (*P* < .001), T stage (*P* = .048), and metastasis (*P* = .009). NNMT levels were higher in tumor stroma of the high group (*P* < .001). N/A: not available.

Similarly, the distribution of NNMT in BC cell populations was analyzed using the SCAR_Atlas_0924 single-cell sequencing dataset (Fig. 5d), and it was found that NNMT was mainly enriched in fibroblasts, endothelial cells, and tumor cells. The baseline characteristics of BC were listed in the Table 2. In the BC clinical cohort (*n* = 90), NNMT protein expression was localized in the cytoplasm of cancer cells and was also expressed in tumor stromal cells, while NNMT expression was low in paracancerous tissues (Fig. 5e). NNMT in tumors and tumor-associated stroma was significantly higher than that in paracancerous tissues. At the same time, NNMT in tumor-associated stroma was higher than that in tumors (Fig. 5f). Moreover, NNMT expression in tumor-associated stroma significantly correlated with molecular typing, TNM, and tumor size of BC (Table 2). These results validated the previous analysis results of the TCGA and PDC databases and suggests that NNMT may be a risk factor for the development of LUAD and BC.

**Table 2:** The clinical characterize of the BC patients in a clinic cohort (*n* = 90).

Clinical parameters	Overall	Low NNMT group	High NNMT group	*P*
***n***	90	54	36	
**Age [mean (SD)]**	52.0 (11.00)	52.8 (11.21)	51.94 (10.39)	.711
**Molecular typing (%)**				.**022**
** A**	7 (7.8)	6 (11.1)	1 (2.8)	
** B**	29 (32.2)	24 (44.4)	5 (13.9)	
** Her2**	8 (8.9)	7 (13.0)	1 (2.8)	
** Basal-like**	45 (50.0)	17 (31.5)	28 (77.8)	
**TNM stage (%)**				.**003**
** I**	12 (13.3)	11 (20.4)	1 (2.8)	
** II**	56 (62.2)	32 (59.3)	24 (66.7)	
** III**	19 (21.1)	9 (16.7)	10 (27.8)	
**T (%)**				.**030**
** T0**	1 (1.1)	1 (1.9)	0 (0.0)	
** T1**	20 (22.2)	17 (31.5)	3 (3.3)	
** T2**	63 (70.0)	31 (57.4)	32 (88.9)	
** T3**	5 (5.6)	4 (7.4)	1 (2.8)	
***N* (%)**				.211
** N0**	45 (50.0)	30 (55.6)	15 (41.7)	
** N1**	44 (48.9)	23 (42.6)	21 (58.3)	
**M (%)**				^a^
** M0**	88 (97.8)	53 (98.1)	35 (97.2)	
** M1**	0 (0.0)	0 (0.0)	0 (0.0)	
**NNMT expression**				
** Stroma of tumor [mean (SD)]**	110.52 (81.42)	53.0 (42.09)	196.81 (37.55)	<.001

Clinical features of 90 breast cancer patients grouped by NNMT expression (low vs. high). Molecular typing (*P* = .022), TNM stage (*P* = .003), and T stage (*P* = .030) differed significantly. High NNMT group showed elevated stromal expression (*P* < .001).

^a^Since the standard deviation of both groups is 0, an independent samples *t*-test cannot be performed.

### 3.6. Prognostic value of stroma NNMT protein expression in LUAD and BC patients

This study further investigated the specific expression patterns of the NNMT protein in tumor tissues, with a particular focus on its expression levels in the tumor stroma and their association with prognosis in patients with LUAD and BC.

**Figure 6:**
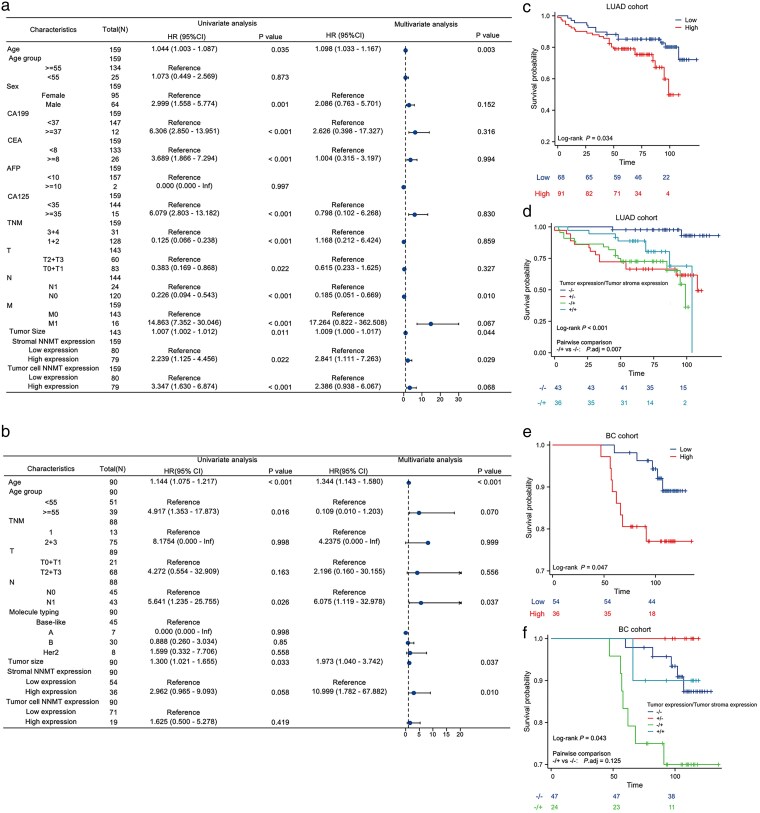
Prognostic analysis of stromal NNMT protein expression in LUAD and BC cohort. (a) Forest plot illustrating univariate and multivariate Cox regression analyses of OS in the LUAD cohort. (b) Forest plot showing univariate and multivariate Cox regression analyses of OS in the breast cancer cohort. (c) Kaplan–Meier survival curves for LUAD patients stratified by high and low NNMT expression in tumor stromal cells. High: NNMT high expression; low: NNMT low expression. (d) Kaplan–Meier survival analysis of LUAD patients categorized into four groups based on combined tumor cell and tumor stromal NNMT expression: “−/−” (low tumor/low stroma), “−/+” (low tumor/high stroma), “+/−” (high tumor/low stroma), and “+/+” (high tumor/high stroma). (e) Kaplan–Meier survival curves for BC patients stratified by high and low NNMT expression in tumor stromal cells. (f) Kaplan–Meier survival analysis of BC patients classified into four groups according to combined tumor cell and stromal NNMT expression patterns.

In the LUAD cohort, univariate Cox regression analysis indicated that overall survival (OS) was associated with age, gender, CA199, CEA, CA125, TNM stage, lymph node metastasis, distant metastasis, tumor size, NNMT expression in the tumor cells, and NNMT expression in the tumor stroma (all *P* < .10; Fig. 6a). Subsequent multivariate Cox regression analysis confirmed that stromal NNMT expression [hazard ratio (HR) = 2.841, 95% confidence interval (CI): 2.111–7.263; *P* = .029] served as an independent risk factor for OS in LUAD patients (Fig. 6a). Kaplan–Meier survival analysis further demonstrated that patients with high stromal NNMT expression had significantly shorter OS than those in the low-expression group (*P* = .034; Fig. 6c). To further elucidate the prognostic implications of NNMT expression patterns, patients were categorized into four groups based on tumor cell and tumor stroma NNMT expression levels: “−/−” (low tumor/low stroma), “−/+” (low tumor/high stroma), “+/−” (high tumor/low stroma), and “+/+” (high tumor/high stroma). Overall comparison revealed significant prognostic differences among the four groups (*P* < .001; Fig. 6d), with the “−/+” group showing significantly worse prognosis compared to the “−/−” group (HR = 7.839, 95% CI: 2.114–29.065; *P* = .007; Fig. 6d).

In the breast cancer cohort, univariate Cox regression analysis similarly identified age, lymph node metastasis, tumor size, and stromal NNMT expression as factors associated with patient OS, while NNMT expression in tumor cells was not (all *P* < .10; Fig. 6b). Multivariate analysis further established stromal NNMT expression (HR = 10.999, 95% CI: 1.782–67.882; *P* = .01) as an independent prognostic risk factor (Fig. 6b). Kaplan–Meier curves also indicated that patients with high stromal NNMT expression had significantly poorer OS (*P* = .047; Fig. 6e). In the four-group combined analysis, although overall prognostic differences were observed (*P* = .043; Fig. 6f), the comparison between the “−/+” and “−/−” groups did not reach statistical significance (HR = 3.537, 95% CI: 1.008–12.411; *P* = .125; Fig. 6f). Nevertheless, a trend toward poorer prognosis was noted in association with high stromal NNMT expression.

In summary, these findings indicate that high stromal NNMT expression correlates with poor outcomes and represents an independent prognostic factor.

## 4. Discussion

Since Otto Warburg found tumor cells consumed glucose and secreted lactate even in the presence of oxygen, metabolic reprogramming of tumor has become a new therapeutic target [37]. However, the exact regulatory mechanisms involved remain largely elusive. Recent studies have uncovered an interesting role of TME in prompting cancer metabolism and behavior, including cancer cell metabolism, interactions between cancer cells and non-cancerous cells, location and heterogeneity of tumors, and so on [38]. Thus, the search for a metabolic regulator involved in TME has received increasing attention.

NNMT is a novel regulator of metabolism in many tissues and tumors. NAM, known as the substrate of NNMT, was the precursor of NAD, which was a cofactor for multiple enzymes of the oxidation of energy sources [39]. However, NNMT was involved in much more than the NAM pathway. As an example, NNMT was upregulated in the liver and adipose tissue of obese and diabetic mice. Knockdown of NNMT prevented diet-induced obesity [40]. Glucose availability regulated NNMT expression via the mTOR signaling pathway [41]. Besides, a hepatic NNMT suppressed *in vivo* altered glucose and cholesterol metabolism, and MNAM, a metabolic product of NNMT, played a significant role in it [42]. In ovarian cancer cells, upregulation of NNMT induced by BRCA1 depletion decreased mitochondrial respiration and reprogrammed metabolism [43]. Consequently, such ubiquity presumably connotes NNMT's important roles in metabolic reprogramming.

In our study, NNMT mRNA was specifically high in the liver, which governed body energy metabolism. The result agreed with findings from the past research that hepatic expression of NNMT was generally most prominent but highly variable [42, 44]. Besides specific expression in the liver, the level of NNMT mRNA in most tumors was higher than that in the adjacent tumor. It arose the potential role of NNMT as a diagnostic target in pan-cancer. We also revealed the prognosis values of NNMT mRNA, as it was related to clinical TNM stage and prognosis indexes (OS and DSS) by using the univariate Cox regression. It has been indicated that NNMT in malignant tumors affected cell migration, metastasis, proliferation [26], and its overexpression correlated to poor prognosis in several solid tumors. Based on the function of NNMT, we examined more metabolic pathways related to NNMT at both the tissue and the cellular level. NNMT was positively related to glycosphingolipid biosynthesis and glycosaminoglycan biosynthesis but negatively related to lipoic acid metabolism, butanoate metabolism, and linoleic acid metabolism. The glycosphingolipid biosynthesis pathway is known to be involved in tumor signal transduction, drug resistance, and the shaping of an immunosuppressive microenvironment. Therefore, we believe that NNMT is likely to play a key role in driving tumor metabolic plasticity and malignant progression by regulating such conserved pathways.

The aforementioned study shed the light on the role of NNMT at the gene level. Whereas the function of NNMT at the protein level probably had different results, which would be precise to reflect the real situation. We analyzed the protein expression and indicated that NNMT was higher in the tumor tissues than in normal or adjacent tissues in almost all cancers, except liver cancer. Similar to mRNA, NNMT protein could still discriminate the prognosis of cancers.

As to the above investigations based on mixed tissues, it remained elusive which kind of cell NNMT played a critical role in. A sequence profile at the single-cell level from TISCH suggested that the expression of NNMT was higher in the stromal cells than in the others. Among them, fibroblast cells expressed the highest NNMT. To validate it, we collected IHC results in the LUAD and BC cohorts, which showed that NNMT expressed more in stroma than in tumor or the adjacent tumor. High stromal NNMT expression was also observed in Eckert et al.'s study, which regulated the phenotype of CAFs via DNA hypomethylation in multiple cancer types [45]. The stromal cells were one of the most important components of TME, though the components varied across cancer types [46]. In conclusion, our study determined that NNMT was a metabolic regulator in TME.

Building upon previous findings from our team, this study further expands the understanding of NNMT as a significant tumor microenvironment molecule and confirms its broad prognostic value across multiple solid tumors. Previous studies by our team demonstrated that NNMT is overexpressed in both tumor and stromal cells, with particularly high expression in the stromal compartment of CRC. Overexpression of NNMT in stroma cells is associated with metastasis and unfavorable survival of CRC patients [13]. Given these established findings, this study did not revisit the analysis of CRC but instead focus on LUAD and BC, systematically analyzing the spatial distribution characteristics of NNMT protein expression and its clinical implications. Initial analysis of public databases confirmed that NNMT shows significant prognostic value at both mRNA and protein levels in LUAD and breast cancer. Furthermore, IHC validation revealed substantial spatial heterogeneity in NNMT expression, with significantly higher levels in both tumor cells and stromal cells compared to adjacent normal tissues in LUAD. This observation aligns with previous reports of NNMT upregulation in NSCLC tissues [47]. More importantly, by systematically comparing expression patterns across different anatomical microenvironments, this study demonstrates that NNMT expression in the tumor stroma possesses significant prognostic value. Specifically, in LUAD, stromal NNMT expression was identified as an independent risk factor for overall survival, with high-expression patients showing significantly shorter survival. A similar trend was observed in the BC cohort, where high stromal NNMT expression served as an indicator of poor prognosis. These findings not only align with existing studies demonstrating that NNMT knockdown inhibits tumor cell proliferation and tumorigenicity [48], but also extend the pro-tumorigenic function of NNMT to the stromal compartment of the tumor microenvironment. This suggests NNMT may contribute to malignant progression through modulation of stromal functions. Collectively, our series of studies provides initial evidence that stromal NNMT holds promise as a pan-cancer prognostic biomarker capable of identifying patient subgroups characterized by highly aggressive and immunosuppressive tumor microenvironments. Building upon this, our future objective is to translate stromal NNMT into a practical prognostic stratification tool to guide individualized adjuvant therapeutic decisions. Definitive validation of this pan-cancer potential, however, will require confirmation in larger, prospective cohorts across a broader spectrum of malignancies.

Beyond its prognostic correlation, our findings prompt speculation on the potential mechanistic roles of stromal NNMT. The consistent prognostic significance of stromal NNMT across CRC, LUAD, and BC suggests a conserved pro-tumorigenic function beyond a mere correlative marker. While the precise mechanisms in LUAD and BC warrant future investigation, insights can be drawn from our prior functional study in CRC [13]. In that work, we demonstrated that NNMT overexpression in the stromal compartment and its enzymatic product, 1-methylnicotinamide (1-MNA), directly enhanced the migration and invasion capabilities of CRC cells. This provides direct evidence that stromal NNMT can actively shape a pro-metastatic TME via a metabolite-mediated paracrine mechanism. Extending this concept to LUAD and BC, and synthesizing with existing literature, we hypothesize that stromal NNMT may drive tumor progression through integrated pathways: (i) metabolic-epigenetic reprogramming: NNMT catalyzes the methylation of nicotinamide, consuming a large amount of S-adenosylmethionine, leading to global hypomethylation in the tumor microenvironment, which in turn activates a series of gene programs related to tumor progression. (ii) Metabolic crosstalk: The NNMT-1-MNA axis or other metabolic alterations might fuel adjacent tumor cells, supporting a metabolic synergy.

In summary, this study deepens the understanding of NNMT as a prognostic biomarker across multiple cancer types and emphasizes the clinical importance of its spatial localization—particularly its enrichment in the tumor stroma. Future research should further explore the specific molecular mechanisms of NNMT within the tumor stroma, providing a theoretical foundation for its potential use as a diagnostic marker or therapeutic target.

## 5. Conclusion

In summary, our study examined NNMT mRNA and protein expression characteristics, correlated pathways, prognostic value, and relationships with TME in LUAD, BC, and CRC. Additionally, we provided several possible clues regarding the mechanism by which NNMT impacted metabolism. NNMT may alter cancer metabolism via its high expression in stromal cells, especially fibroblasts within the TME. In order to further determine this, NNMT-related CAFs and other cells in TME may as well warrant further investigation. In addition, this study established clinical cohorts of LUAD and BC, verified the high expression characteristics of NNMT in stromal cells, and established a prognostic model for LUAD and BC based on NNMT protein expression, further elucidating the value of stromal NNMT as an independent prognostic biomarker for both LUAD and BC.

## Supplementary Material

bgag020_Supplementary_Data

## Data Availability

Public data are available in a public, open access repository on the online website, which are listed in the methods. The specific sample and dataset identifiers (e.g. from TCGA, PDC, and CPTAC) used in this study have been comprehensively listed in Supplementary Table. In-house data information and R custom scripts for analyzing data are available upon reasonable request.
