# Supplementary material for: Stromal NNMT overexpression as an independent prognostic biomarker in lung adenocarcinoma and breast carcinoma
Source: Carcinogenesis. 2026 Mar 30;47(2):bgag020. doi: 10.1093/carcin/bgag020 (PMC13174943; doi:10.1093/carcin/bgag020)
Supplement: bgag020_Supplementary_Data [file bgag020_supplementary_data.zip › Supplemental Figure.docx]

**
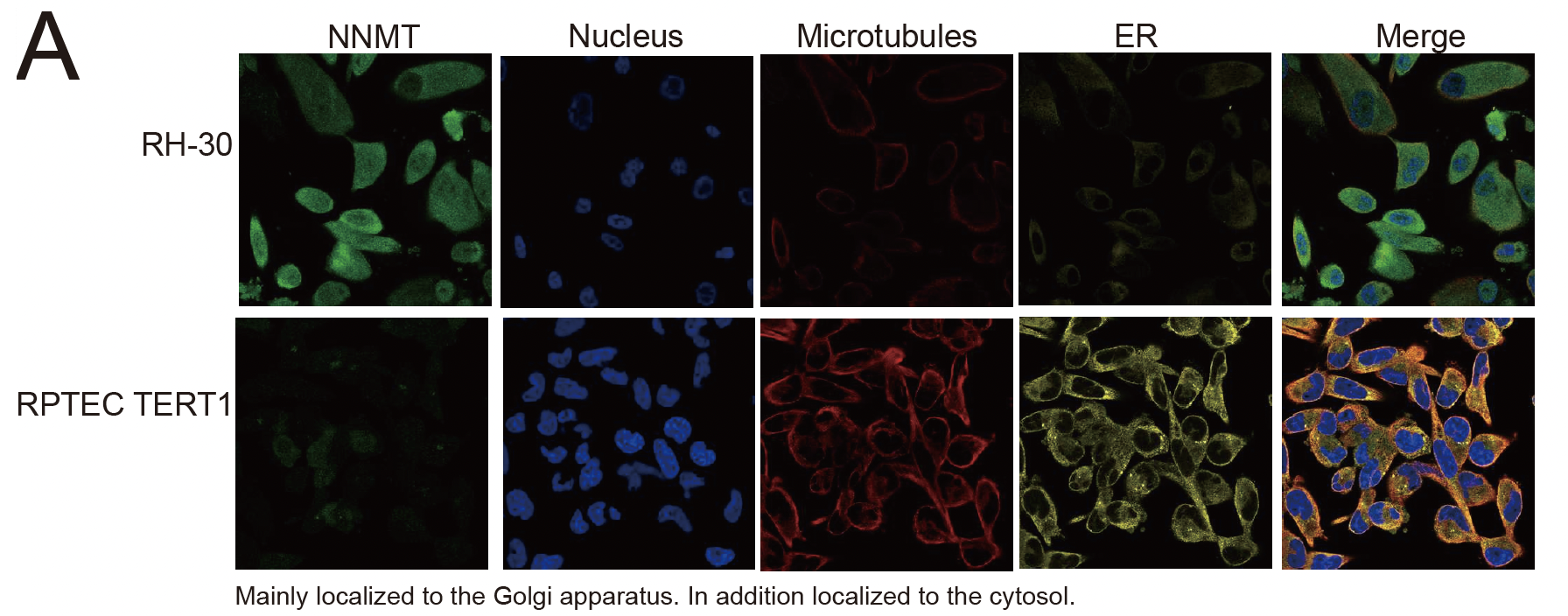
Figure 1.** **The protein features of NNMT in HPA** . A) Immunofluorescence of NNMT protein in RH-30 and RPTEC/TERT1 cell lines from the HPA database.
